# Cellars

**Published:** 1874-12

**Authors:** 


HALL'S

### JOURNAL OF HEALTH

AND

MISCELLANY.

VOL. XXI.—DECEMBER, 1874.—NO. XII.

### CELLARS.

WITHIN a year or two, and even within three or four months, several of our most wealthy citizens have died, not old, in fact in their prime, without any satisfactory cause. They were in usual health not long before, but began to complain of not feeling very well; they lost their appetite, their activity and accustomed vivacity; concluded they had somehow or other "taken a little cold," and while hoping that it would soon pass off, as "little colds" had done a thousand times before, graver symptoms manifested themselves, running into typhoid fever, and soon the end came.

In the upper part of the city a family of six or eight persons lived in country style, cultivating a garden patch; the water supply was taken from a "natural spring," literally as clear as crystal, at the foot of the hill. They had lived there, in health, for years; yet one by one the members paled away, sickened, and some of them died. The physician in charge was satisfied there was some hidden cause which it was his duty to ferret out, if possible. He first directed his attention to the "crystal fountain," led thereto from some "improvements" which were making in the neighborhood. On allowing a glass of water to settle, a considerable amount of sediment was deposited, which, on microscopical examination first, and then a careful chemical analysis, it was satisfactorily ascertained that the sediment from the limpid spring was identical with human excrement; an old privy sink having been opened and a small drain finding its way to a spot just above the spring, and sinking down. I saw a lady at Long Branch last summer, a mother who had lost four children, who had come to New York a few weeks before in perfect health from the country. They rented an elegant furnished house. The carpets had not been taken up for five or six years; this, connected with some dampness in the building, generated an atmosphere perfectly poisonous to little children, inducing diphtheria. It has been often noticed that men have builded dwellings for themselves, and soon after taking possession have sickened and died, sometimes from newly laid lead pipes, at others from damp walls, and then again from poisonous green papering. In cases not a few, the whole family has sickened from the house having been built on made ground, a wet soil, a low spot, into which foul matters have been washed and settled for years, then being covered up chemical decomposition has followed, throwing out from beneath foul gases, which, working up their way through the filled earth, and through the cellar floor up into all the rooms, have wrought their baleful influence.

Most of our city mansions have the furnace in the cellar, and now that winter is upon us the cellar is made the warmest place in the house, at least warm enough to promote the decomposition of many of the things which households consign to that part of the building, making it, in fact, a common receptacle for all that is old, dilapidated and worn out; to say nothing of decayed vegetables, bones, kitchen scraps, rotting carpets, door mats, shoes and such like. As the cellar door opens up into the dwellings, and is ajar a greater part of the time, and often is left wide open all night by the carelessness of servants, every habitable apartment in the whole building has its atmosphere saturated with the foul emanations from below, and there is no health in the house; which is very much wondered at, as the bracing cold weather has come, besides the advantages of a seaside residence during the summer, which had been calculated upon as giving a new lease of health and life for months to come. Every nook and corner of a cellar ought to be kept as clean as a dining room or parlor. There are not many such in all New York; there is one, at least, that of our well-known and most estimable fellow-citizen, Homer Morgan, Esq. Having had occasion to go into it the other day, what is said is after personal inspection. It contains three or four gaslights; it is most thoroughly whitewashed two or three times a year; there was not a chip or lump of coal on the whole floor. The kindling wood was carefully piled up in one department; the different kinds of coal, soft and hard, was stowed away in their separate bins; there was not a spider's web anywhere discoverable; everything seemed to be placed on shelving adapted to its purpose; the only use of the floor was to be walked on, with access both from the in and outside of the building. If there is an architect in the whole city of New York who understands his business hygienically, Mr. Barnum should make money by showing him up at the Hippodrome. In this model cellar was one arrangement which, to our mind, was nothing less than an intellectual delight. The soil-pipe was large, made of iron, and was in full view against the side of the, literally, snow-white wall, so that if there was a leakage of the size of a single, solitary drop, it could not but be seen at once, for this soil pipe was whitewashed, as white as the driven snow, and a single drop would have caused a considerable splotch of discoloration. In almost all dwellings, the soil pipe is under the cellar floor, or plastered over against the wall, making the discovery of a leakage almost impossible, until its accumulation should burst its bounds and break out in an unendurable stench. No comments or enlargements are needed on this statement of facts. One suggestion only is made as an improvement on the soil pipe idea; let all water-closets be in the rear corner of the building, and let the pipes discharge themselves immediately through the wall into an iron leader on the outside, to discharge in turn into sewers in the back yards leading into the street sewers, without passing under any human habitation; this can be done, and ought to be done, by imperative legal enactment, on the principle of its being for the public good; or the trouble and expense would be much less if the leaders were in the front of the house, then the contents of all the sinks and water-closets could be discharged directly into them, and conveyed at once into the city sewers under the street, and not pass under the whole extent of the cellar floor. No fact is more thoroughly established, in connection with health and disease, than that diphtheria is the result of bad air and uncleanly surroundings.

